# Rural-urban disparity in cancer burden and care: findings from an Indian cancer registry

**DOI:** 10.1186/s12885-024-12041-y

**Published:** 2024-03-06

**Authors:** Divya Khanna, Priyanka Sharma, Atul Budukh, Rajesh Vishwakarma, Anand N. Sharma, Sonali Bagal, Varsha Tripathi, Vijay Kumar Maurya, Pankaj Chaturvedi, Satyajit Pradhan

**Affiliations:** 1 Present Address: Department of Preventive Oncology and Varanasi Cancer Registry, Mahamana Pandit Madan Mohan Malaviya Cancer Centre (MPMMCC) and Homi Bhabha Cancer Hospital (HBCH), Tata Memorial Centres, 221005 Varanasi, Uttar Pradesh India; 2 Centre for Cancer Epidemiology, Tata Memorial Centre, Homi Bhabha National Institute, 400012 Mumbai, India; 3 Department of Surgical Oncology, Homi Bhabha National Institute, Training School Complex, Anushakti Nagar, 400094 Mumbai, India; 4 Department of Radiation Oncology and Director, Mahamana Pandit Madan Mohan Malaviya Cancer Centre (MPMMCC) and Homi Bhabha Cancer Hospital (HBCH), Tata Memorial Centres, 221005 Varanasi, Uttar Pradesh India

**Keywords:** Registries, Disparity, Incidence, Mortality, Cancer, Low-and middle-income countries

## Abstract

**Background:**

Cancer incidence and mortality vary across the globe, with nearly two-thirds of cancer-related deaths occurring in low- and middle-income countries. The rural-urban disparity in socio-demographic, behavioural, and lifestyle-related factors, as well as in access to cancer care, is one of the contributing factors. Population-based cancer registries serve as a measure for understanding the burden of cancer. We aimed to evaluate the rural-urban disparity in cancer burden and care of patients registered by an Indian population-based cancer registry.

**Methods:**

This study collected data from Varanasi, Uttar Pradesh, India, between 2017 and 2019. Sex and site-specific age-standardised rates for incidence and mortality per 100,000 population were calculated. Rural-urban disparities in cancer incidence and mortality were estimated through rate differences and standardised rate ratios (with 95% confidence intervals). Univariable and multivariable regressions were applied to determine any significant differences in socio-demographic and cancer-related variables according to place of residence (rural/urban). Crude and adjusted odds ratios with 95% confidence intervals were calculated.

**Results:**

6721 cancer patients were registered during the study duration. Urban patients were older and had better literacy and socioeconomic levels, while rural patients had higher odds of having unskilled or semi-skilled professions. Diagnostic and clinical confirmation for cancer was significantly higher in urban patients, while verbal autopsy-based confirmation was higher in rural patients. Rural patients were more likely to receive palliative or alternative systems of medicine, and urban patients had higher chances of treatment completion. Significantly higher incidence and mortality were observed for oral cancer among urban men and for cervical cancer among rural women. Despite the higher incidence of breast cancer in urban women, significantly higher mortality was observed in rural women.

**Conclusions:**

Low- and middle-income countries are facing dual challenges for cancer control and prevention. Their urban populations experience unhealthy lifestyles, while their rural populations lack healthcare accessibility. The distinctness in cancer burden and pattern calls for a re-evaluation of cancer control strategies that are tailor-made with an understanding of urban-rural disparities. Context-specific interventional programmes targeting risk-factor modifications, cancer awareness, early detection, and accessibility to diagnosis and care are essential.

**Supplementary Information:**

The online version contains supplementary material available at 10.1186/s12885-024-12041-y.

## Background

Globally, cancer is one of the leading causes of mortality; two-thirds of these deaths occur in low- and middle-income countries [1]. Moreover, large variations are reported in cancer incidence, patterns, and mortality among different regions of a country [2]. The disparity in the continuum of cancer care, especially among rural populations, has significantly contributed to this disproportion globally [3]. India is a culturally diverse country, with two-thirds of its population (833 million) residing in rural regions and displaying large regional and rural-urban variations in lifestyles, mortality, and morbidity rates [4, 5]. Moreover, rural areas continue to suffer from challenges related to inadequate accessibility, affordability of healthcare, and underutilization, compounded by the absence of robust health information systems. In contrast, urban regions have witnessed significant improvements in these aspects [3, 6]. A staggering majority (80%) of the elderly with unmet healthcare needs are concentrated in the rural regions of India [5]. Lifestyle and behavioural risk factors are also increasing, especially in urban areas, leading to an epidemiological transition in the country.

Due to the lack of organized health information systems and weak cause-of-death registration systems, population-based cancer registries (PBCRs) serve as a measure for understanding the state and national-level burden of cancers in India and are recognised as vital components of national cancer control programmes [7]. However, there is currently an urban predominance in cancer coverage by cancer registries [8]. Hence, to understand the rural-urban disparity in cancer burden and care, registry-based studies from Indian settings that are predominantly rural are needed.

The Government of India (GOI) is committed to universal health care coverage, which requires the identification of disparities, their drivers, and the mitigation of them through targeted policy interventions [9]. Therefore, we aimed to study the rural-urban disparity in burden and care of over 6,000 patients who were registered by the PBCR in an Indian setting between the years 2017 and 2019 so that future cancer control planning in the country will be more considerate of the existing urban-rural differences.

## Methods

### Study settings and population

#### Varanasi district

**Fig. 1:**
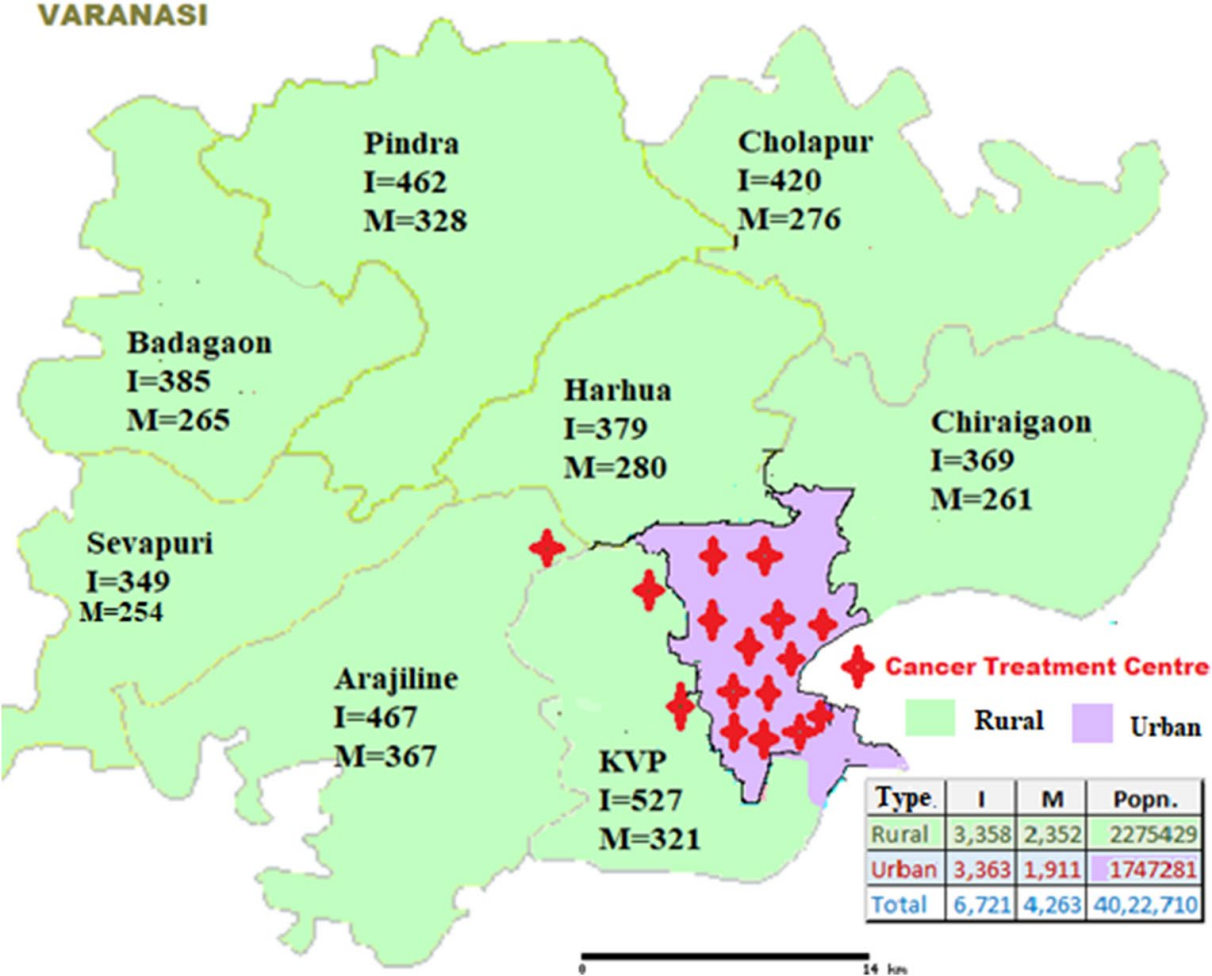
Distribution of cancer incidence (I), mortality (M) and tertiary cancer treatment centres in Varanasi district, Uttar Pradesh, India

The estimated population of Varanasi is 4 million (40,05,176), with rural predominance (57%), in an area of 1535 square km. The rural-urban classification is based on the Census of India. The district has eight rural blocks with 1295 villages and 90 urban wards, which are not adequately covered by conventional cancer screening programmes. There is an established three-tier healthcare delivery and referral system as per the National Health Mission of the GOI. The district has three government-supported tertiary cancer care centres (two TCCs and one apex medical college) and a few private centres equipped with cancer-related diagnostic and treatment facilities, all of which are concentrated in the urban parts of the district. The rural residents need to travel at least 6 to 24 miles to reach these TCCs, and have limited transportation facilities. (Fig. 1) [7, 10].

#### Varanasi population-based cancer registry

The Varanasi PBCR was established in 2017, as a part of PBCRs operated by the Tata Memorial Centres (TMC). It provides representative statistics for cancer burden in the Uttar Pradesh state of Northern India, which is the most populous state of India and is predominantly rural with poor health indicators. The TMC has an agreement with the district health administration to conduct cancer registration. Through an active registration process, data on cancer cases are collected from various sources in the district through a pre-defined proforma and entered into the CanReg5 software of the International Agency for Research on Cancer (IARC). Field investigators were trained in data extraction and entry methods, and are periodically monitored by the faculty of the Centre for Cancer Epidemiology, TMC [7, 10]. Quality control was ensured through systematic and random checks, duplication removal, re-abstraction of 5% of randomly selected cases, retraining of the staff, and calculating data quality indices for completeness. (Supplementary Table 1) [11, 12].

### Urban-rural definition

As per the Census (2011), India’s classification for urban regions includes:

(i) All places with a municipality, corporation, cantonment board, or notified town area committee,
(ii) All other places that satisfy the following criteria: (a) minimum population of 5000; (b) at least 75% of the working male population engaged in non-agricultural pursuits; (c) a population density of at least 400 persons per square kilometre [13].

### Statistical analysis

Cancer incidence and mortality data from 2017 to 2019 were obtained from the PBCR, Varanasi district of Uttar Pradesh state of India. The extracted data included demographic information for the age, gender, place of residence, religion, education, mother tongue, occupation, and monthly income. The tumour details included topography and morphology of the primary site of cancer, basis of diagnosis, type of treatment taken, treatment status, and the outcome in the form of death of the patient. Malignancies were classified according to the International Classification of Diseases for Oncology, Version III (ICD-O) [7, 10]. 

We calculated the sex and site-specific cancer burden through crude and age-adjusted rates (AAR) for incidence (AAIR) and mortality (AAMRs) per 100,000 population and cumulative risk (probability that an individual will be diagnosed with cancer for 0–74 years of age group) for rural and urban regions. The AARs were computed by using the direct standardisation method with the World Standard Population 2000 as a reference. The rural-urban disparities in cancer incidence and mortality were quantitatively assessed with two disparity measures; Rate difference (RD; AAR of rural population ─ AAR of urban population) [14] and Standardised Rate Ratio (SRR; AAR of rural population **/**AAR of urban population, with 95% confidence intervals) [15]. Univariable and multivariable regressions were used to assess any significant difference in the socio-demographic and cancer-related variables according to the place of residence (rural/urban). Variables found significant on univariable analysis (p-value < 0.2) [16] were entered into the multivariable regression model after excluding collinearity. Crude and adjusted odds ratios (OR) with a 95% confidence interval (CI) were calculated. The level of statistical significance for the multivariable regression was set at a p-value of less than 0.05. The data were entered into MS Excel and analysed using the Statistical Package for Social Sciences (SPSS, version 21).

### Patient and public involvement

Patients or the public were not involved in the design, conduct, reporting, or dissemination plans of our research.

## Results

### Socio-demographic profile of patients with cancer

Out of 6721 patients registered under the Varanasi PBCR (2017–2019), 2.3% (156) were in the paediatric age group (0–14 years) and were excluded from further analysis. Among 6565 adult patients, 73.8% (4848) were 45 years or older, more than half were males (3670, 55.9%), and one-fifth were illiterate (1370, 20.9%). Most of the study participants were Hindus (5774, 88.0%) by religion and had Hindi as their mother tongue (6099, 92.9%) (Supplementary Table 2).

### Cancer burden

The leading cancer sites in men were the mouth, tongue, trachea bronchus and lung, prostate, liver, and gallbladder, with AAIR as 19.1, 5.3, 3.6, 3.4, 3.4, 3.4 per 100,000 respectively. For women, the leading cancer sites were the breast, cervix, gallbladder, ovary, mouth, and liver, with AAIR as 13.7, 8.4, 7.3, 3.6, 2.5, 2.5 per 100,000 respectively. The overall leading primary sites are given in Supplementary Table 3. The lifetime risk for developing cancer (0–74 years) was the highest for mouth and breast among men and women, respectively. Rural-urban comparisons for cumulative risk for some of the leading sites are given in Supplementary Table 3.

### Rural-urban disparities

On multivariable analysis, older cancer patients (45 years and older) had about 1.5 to 2 times higher odds of belonging to an urban area compared to the rural area. Similarly, the odds of literacy were higher in urban patients than in rural patients. Patients using non-Hindi dialect were higher in rural patients compared to their urban counterparts. Also, the proportion of farmers, unskilled, semi-skilled, and skilled workers was lower among urban patients, but that of professional and semi-professional workers was higher when compared to rural patients. The odds of being in lower middle, and upper and upper middle socio-economic class were 1.4 times higher among urban patients than rural patients. Diagnostic and clinical record confirmation for cancer diagnosis was significantly higher in urban patients, while verbal autopsy confirmation was higher in rural patients. Furthermore, we observed that the odds of receiving palliative or alternative systems of medicine were significantly higher for rural patients compared to their urban counterparts. Additionally, the odds of treatment completion were significantly higher for urban patients. A significantly higher proportion of patients were alive at the time of follow-up among urban residents compared to the rural ones. (Table 1)

**Table 1:** Rural-urban differences in the distribution of adult patients with cancer (*N* = 6565)

Variable	Rural (*n* = 3220)	Urban (*n* = 3345)	Crude		Adjusted	
			OR (95%CI)	p-value	OR (95%CI)	p-value
**Age groups (completed years)**						
15–29	206 (53.2)	181 (46.8)	Reference		Reference	
30–44	649 (48.8)	681 (51.2)	1.2 (0.9–1.5)	0.125	1.3 (1.01–1.7)	0.040*
45–59	1237 (50.9)	1195 (49.1)	1.1 (0.9–1.4)	0.387	1.5 (1.2–1.9)	0.002*
≥ 60	1128 (46.7)	1288 (53.3)	1.3 (1.0-1.6)	0.017*	2.0 (1.5–2.6)	< 0.01*
**Sex**						
Female	1472 (50.8)	1423 (49.2)	Reference		Reference	
Male	1748 (47.6)	1922 (52.4)	1.1 (1.0-1.2)	0.010*	0.9 (0.7–1.1)	0.284
**Educational qualification**						
Illiterate	852 (62.2)	518 (37.8)	Reference		Reference	
Literate	811 (55.1)	660 (44.9)	1.3 (1.1–1.5)	< 0.01*	1.3 (1.1–1.5)	0.003*
Up to secondary	1200 (45.7)	1426 (54.3)	1.9 (1.7–2.2)	< 0.01*	1.7 (1.5-2.0)	< 0.01*
Senior secondary or higher	335 (32.8)	686 (67.2)	3.4 (2.8-4.0)	< 0.01*	2.4 (2.0–3.0)	< 0.01*
No information/unknown	22 (28.6)	55 (71.4)	4.1 (2.5–6.8)	< 0.01*	2.8 (1.5-5.0)	0.001*
**Religion**						
Hindu	3054 (52.9)	2720 (47.1)	Reference		Reference	
Others	166 (21.0)	625 (79.0)	4.2 (3.5-5.0)	< 0.01*	6.5 (5.3–7.9)	< 0.01*
**Mother tongue**						
Hindi	2874 (47.1)	3225 (52.9)	Reference		Reference	
Others	346 (74.2)	120 (25.8)	0.3 (0.2–0.4)	< 0.01*	0.3 (0.2–0.4)	< 0.01*
**Occupation**						
Unemployed, student, house-wife	1388 (51.8)	1290 (48.2)	Reference		Reference	
Farmer, skilled worker, semi-skilled worker, unskilled worker, others	1507 (55.8)	1192 (44.2)	0.8 (0.8–0.9)	0.003*	0.7 (0.6–0.9)	0.004*
Profession, semi-professional, clerical, government employee, private employee	319 (27.9)	824 (72.1)	2.8 (2.4–3.2)	< 0.01*	1.8 (1.4–2.3)	< 0.01*
No information	6 (13.3)	39 (86.7)	7.0 (2.9–16.6)	< 0.01*	4.2 (1.7–10.7)	0.002*
**Monthly Income**						
Lower	1693 (58.4)	1208 (41.6)	Reference		Reference	
Lower middle	964 (42.8)	1289 (57.2)	1.9 (1.7–2.1)	< 0.01*	1.4 (1.3–1.6)	< 0.01*
Upper and upper middle	320 (34.9)	596 (65.1)	2.6 (2.2-3.0)	< 0.01*	1.4 (1.2–1.7)	< 0.01*
No information/unknown	243 (49.1)	252 (50.9)	1.4 (1.2–1.7)	< 0.01*	1.1 (0.9–1.4)	0.226
**Basis of diagnosis**						
Clinical	207 (38.4)	332 (61.6)	Reference		Reference	
DCO	9 (25.0)	27 (75.0)	1.9 (0.9-4.0)	0.113	2.4 (1.03–5.6)	0.041*
Radiology	312 (47.7)	342 (52.3)	0.7 (0.5–0.9)	< 0.01*	0.8 (0.6-1.0)	0.052
Cytology	345 (49.7)	349 (50.3)	0.6 (0.5–0.8)	< 0.01*	0.7 (0.5–0.9)	0.005*
Verbal autopsy	835 (75.8)	266 (24.2)	0.2 (0.1–0.2)	< 0.01*	0.3 (0.2–0.3)	< 0.01*
Histology of primary	1512 (42.7)	2029 (57.3)	0.8 (0.7-1.0)	0.060	0.8 (0.7-1.0)	0.065
**Treatment**						
No treatment	168 (50.9)	162 (49.1)	Reference		Reference	
Surgery	242 (45.7)	288 (54.3)	1.2 (0.9–1.6)	0.134	0.9 (0.7–1.3)	0.817
RT	86 (46.2)	100 (53.8)	1.2 (0.8–1.7)	0.308	0.9 (0.6–1.4)	0.782
CT	499 (49.0)	520 (51.0)	1.1 (0.8–1.4)	0.540	0.9 (0.7–1.2)	0.704
Multi-modality	1084 (43.4)	1414 (56.6)	1.3 (1.1–1.7)	0.010*	1.0 (0.8–1.4)	0.614
Other alternative system	154 (64.2)	86 (35.8)	0.6 (0.4–0.8)	0.002*	0.7 (0.4–0.9)	0.045*
Palliative	766 (60.9)	492 (39.1)	0.7 (0.5–0.8)	0.001*	0.7 (0.5–0.9)	0.020*
No information/unknown	221 (43.8)	283 (56.2)	1.3 (1.0-1.7)	0.046*	1.0 (0.7–1.4)	0.828
**Treatment status**						
Not completed	1522 (56.1)	1190 (43.9)	Reference		Omitted due to collinearity	
Complete	627 (42.8)	838 (57.2)	1.7 (1.5–1.9)	< 0.01*		
Ongoing	611 (44.2)	770 (55.8)	1.6 (1.4–1.8)	< 0.01*		
Not applicable	168 (50.9)	162 (49.1)	1.2 (0.9–1.5)	0.072		
No information/unknown	292 (43.1)	385 (56.9)	1.7 (1.4-2.0)	< 0.01*		
**Status**						
Alive	946 (39.9)	1423 (60.1)	Reference		Reference	
Dead	2274 (54.2)	1922 (45.8)	0.6 (0.5–0.6)	< 0.01*	0.7 (0.7–0.8)	< 0.01*

*Significant association

DCO- death certificate only, RT- Radiotherapy, CT- Chemotherapy

### Rate differences for incidence and mortality of leading cancer sites

**Fig. 2:**
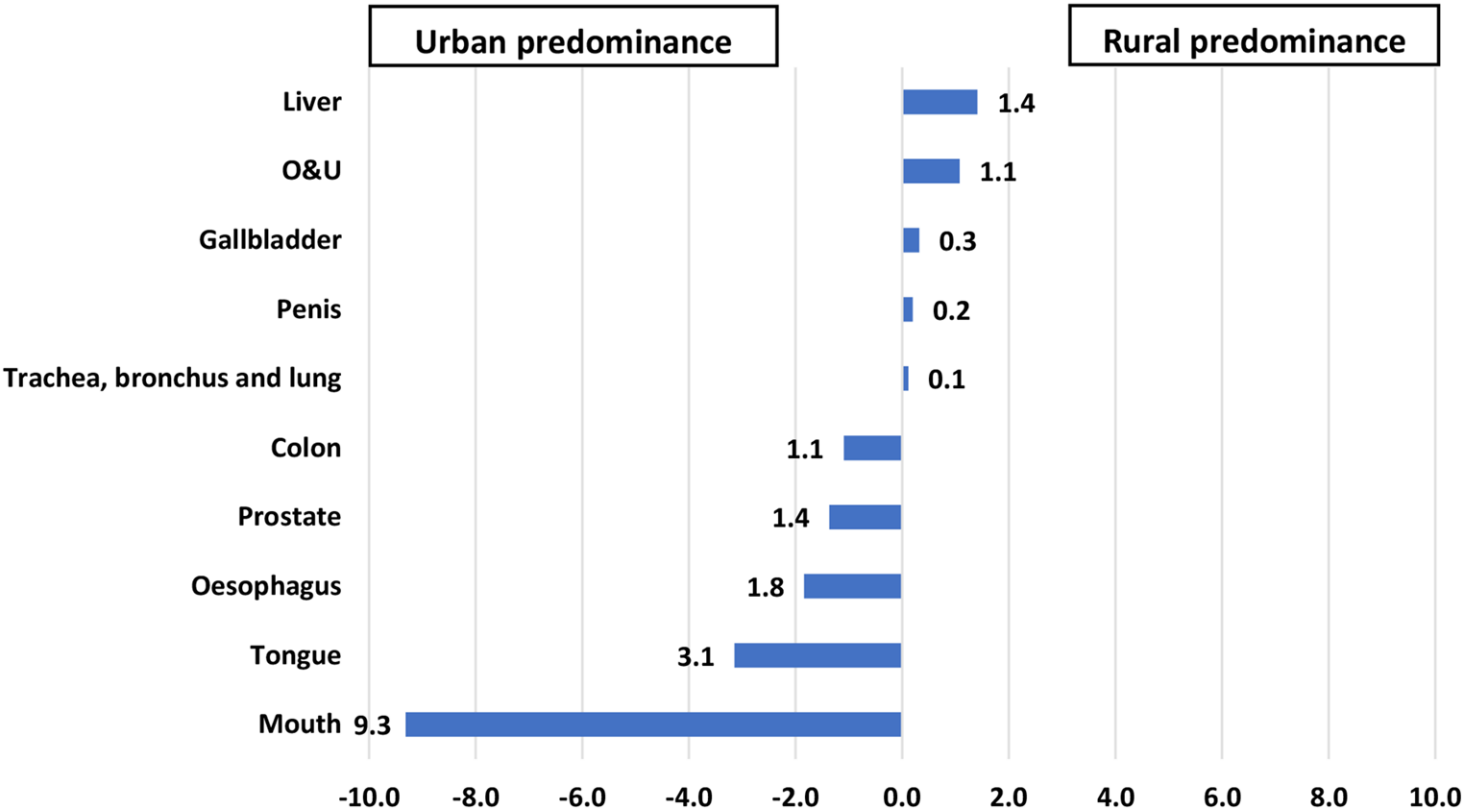
Rate differences in cancer incidence among male patients with cancer residing in rural versus urban areas of Varanasi, 2017–2019. (*N* = 3785)

**Fig. 3:**
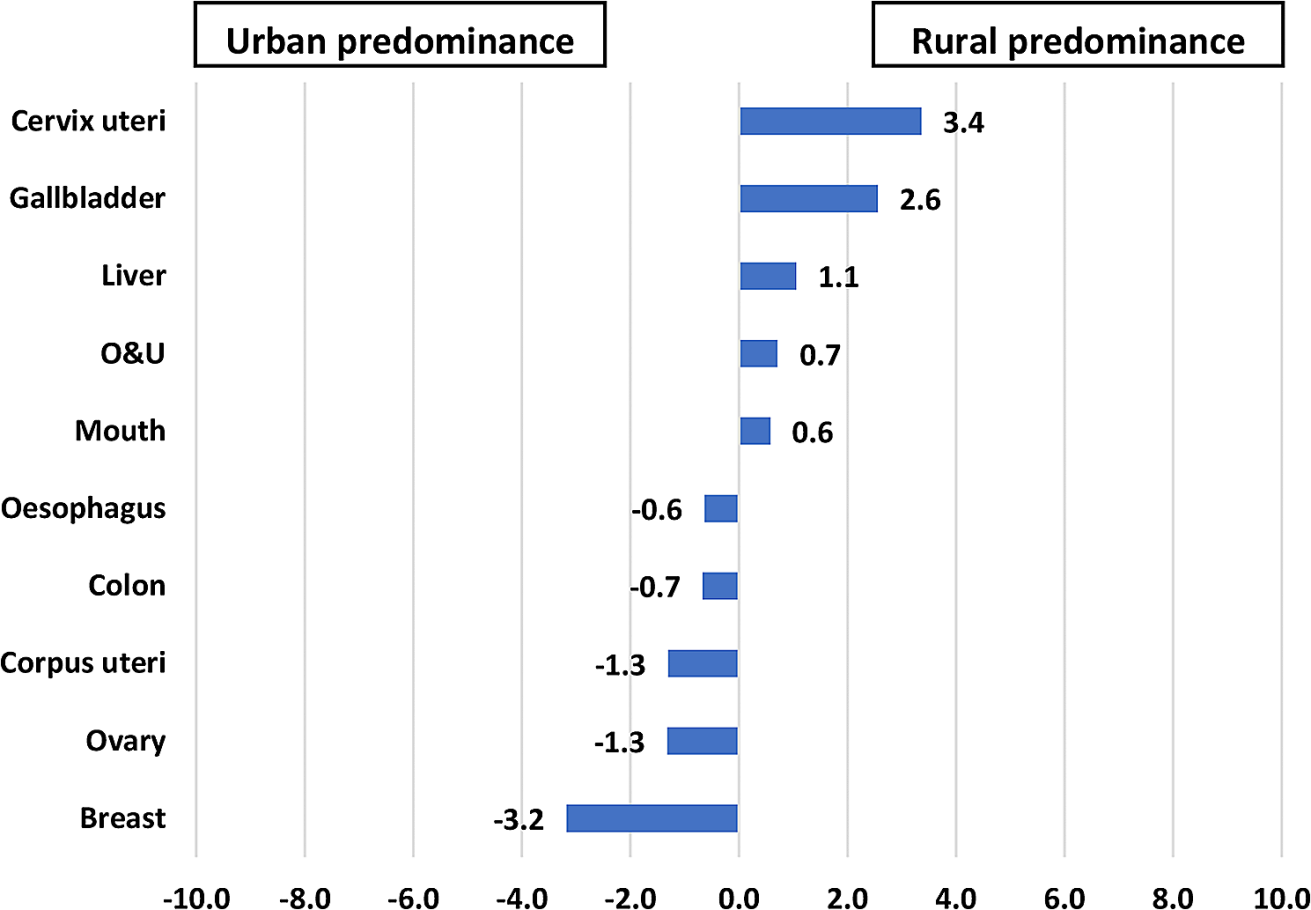
Rate differences in cancer incidence among female patients with cancer residing in rural versus urban areas of Varanasi, 2017–2019. (N = 2936)

The incidence rates of liver, other and unspecified sites (O&U), gallbladder, penis, trachea, bronchus, and lung cancers were higher in rural males, while incidence rates of mouth, tongue, oesophagus, prostate, and colon cancers were higher in urban males. Rural women had higher incidence rates of cervix uteri, gallbladder, liver, O&U, and mouth cancer and urban women had higher incidence rates of breast, ovary, corpus uteri, colon, and oesophageal cancer (Figures 2 and 3).

**Fig. 4:**
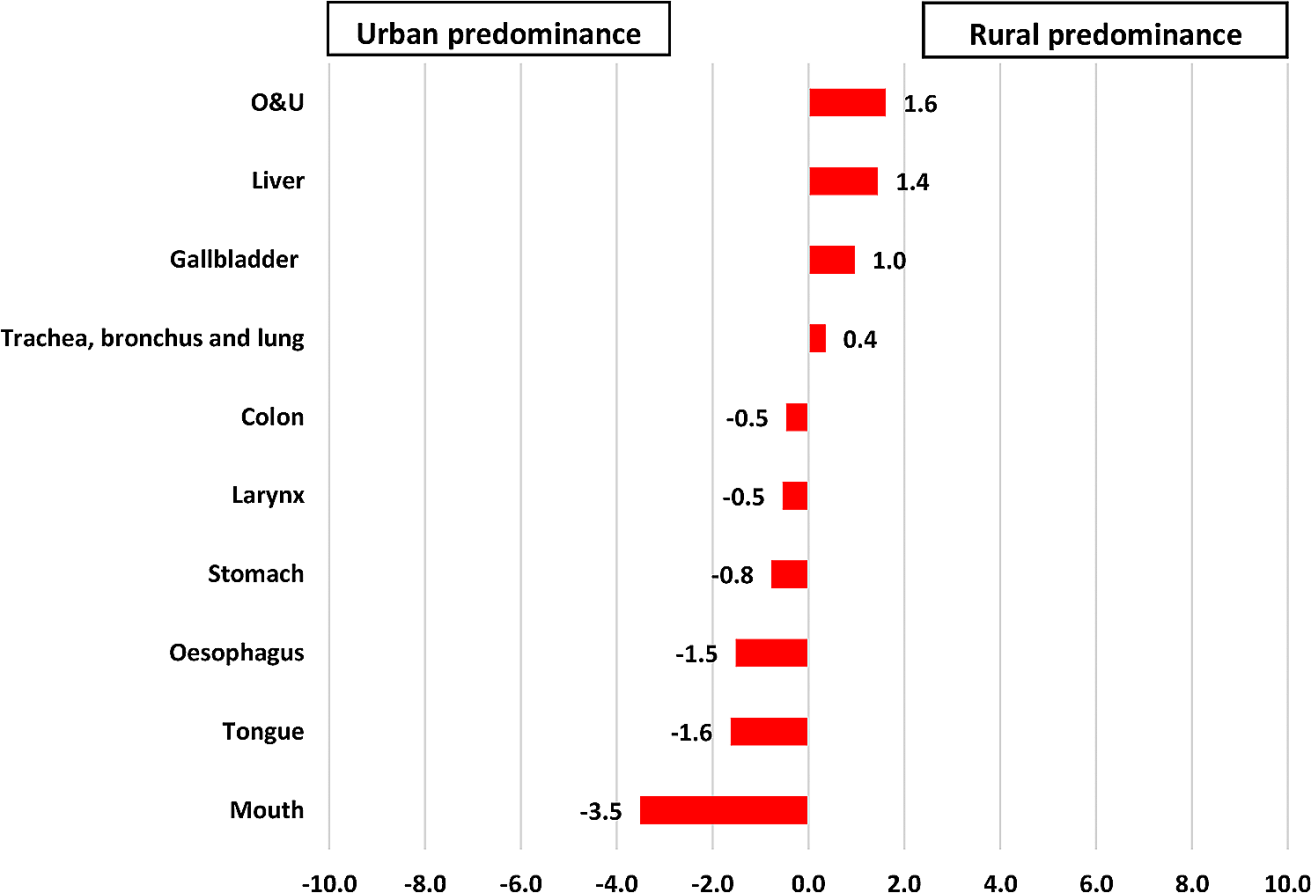
Rate differences in cancer mortality among male patients with cancer residing in rural versus urban areas of Varanasi, 2017–2019. (N = 3785)

**Fig. 5:**
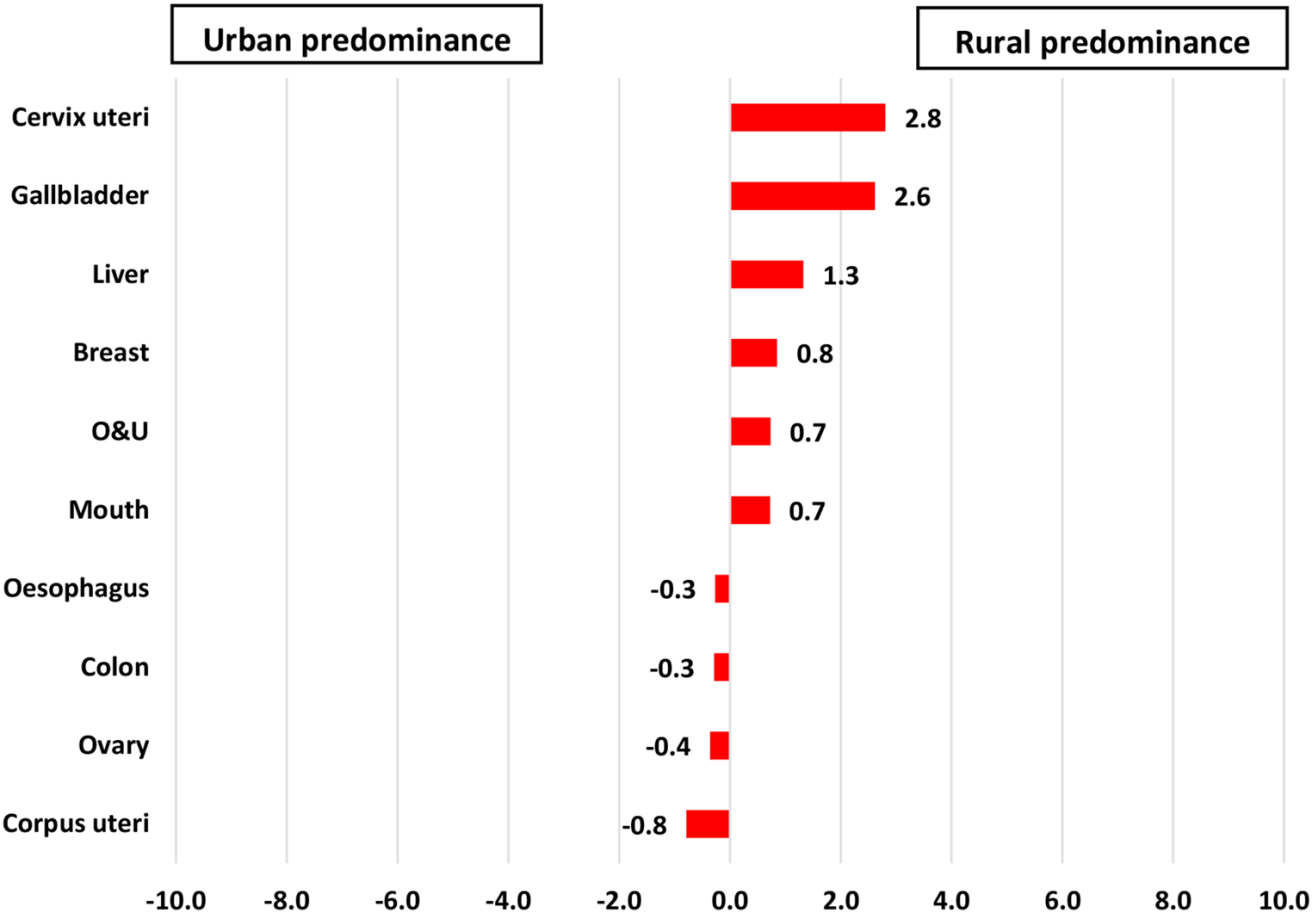
Rate differences in cancer mortality among female patients with cancer residing in rural versus urban areas of Varanasi, 2017–2019. (*N* = 2936)

Similar trends were observed in mortality, with rural males having higher mortality from O&U, liver, gallbladder, trachea, bronchus, and lung cancer and urban males having higher mortality from mouth, tongue, oesophagus, stomach, larynx, and colon cancer. Women living in rural areas had higher mortality from cervix uteri, gallbladder, liver, breast, O&U, and mouth cancer and those living in urban areas had greater mortality from corpus uteri, ovary, colon, and oesophagus cancer (Figures 4 and 5).

### Standardised risk ratios for incidence and mortality of leading cancer sites

We observed a significantly lower all-site cancer incidence (about 25%) and mortality (about 20%) among rural men compared to urban men. No substantial difference in all-site cancer incidence was observed between urban and rural women; however, mortality was about 20% higher among rural women. Rural men had 60–65% lower incidence and 50–60% lower mortality for oesophagus and colon cancers. Also, rural men had 40–45% lower incidence and 25–40% lower mortality for tongue and mouth cancers. Despite about a 35% lower incidence of prostate cancer in rural men, we observed a minimal difference in mortality. Compared to urban men, rural men had about a 50% higher incidence of liver cancer, a 10% higher incidence of gallbladder cancer, and about 45–65% higher mortality for these cancers (Table 2).

**Table 2:** Standardised rate ratio for incidence and mortality of the leading cancer sites among the rural vs. urban population, Varanasi, India, 2017–2019. (*N* = 6721)

Cancer sites	Incidence rate (AAR per 100,000)				Standardised Rate Ratio (95% Confidence Interval)			
	Rural		Urban		Male		Female	
	Male	Female	Male	Female	Incidence	Mortality	Incidence	Mortality
All sites	60.8	59.0	81.7	63.2	0.74 (0.73–0.75) *	0.79 (0.77–0.80) *	0.93 (0.92–0.95) *	1.18 (1.16–1.21) *
Mouth (C03-06)	15.0	2.9	24.3	2.3	0.62 (0.60–0.63) *	0.74 (0.72–0.77) *	1.25 (1.16–1.35) *	1.53 (1.40–1.67) *
Tongue (C01-02)	3.9	1.4	7.1	1.5	0.56 (0.53–0.59) *	0.60 (0.56–0.64) *	0.97 (0.88–1.06)	1.02 (0.91–1.15)
Breast (C50)	0.1	12.7	-	15.9	-	-	0.80 (0.77–0.83) *	1.13 (1.08–1.19) *
Cervical (C53)	-	10.3	-	6.9	-	-	1.49 (1.43–1.55) *	1.74 (1.65–1.83) *
Ovary (C56)	-	3.2	-	4.5	-	-	0.70 (0.66–0.75) *	0.83 (0.76–0.91) *
Corpus uteri (C54)	-	0.6	-	1.9	-	-	0.32 (0.28–0.36) *	0.09 (0.07–0.12) *
Prostate (C61)	2.8	-	4.1	-	0.67 (0.63–0.71) *	0.96 (0.90–1.03)	-	-
Colon (C18)	0.6	0.3	1.7	0.9	0.35 (0.31–0.39) *	0.46 (0.39–0.53) *	0.28 (0.23–0.33) *	0.42 (0.34–0.52) *
Oesophagus (C15)	1.3	0.7	3.1	1.3	0.41 (0.38–0.45) *	0.41 (0.37–0.44) *	0.51 (0.45–0.58) *	0.66 (0.57–0.77) *
Liver (C22)	4.1	3.0	2.7	1.9	1.53 (1.44–1.63) *	1.64 (1.53–1.75) *	1.55 (1.43–1.67) *	1.82 (1.68–1.97) *
Gallbladder (C23-24)	3.5	8.7	3.2	6.2	1.10 (1.04–1.17) *	1.45 (1.35–1.55) *	1.42 (1.35–1.48) *	1.52 (1.45–1.60) *

*Significant difference

Compared to urban women, rural women had about 50% or higher incidence of cervical, liver, and gallbladder cancer and 50% or lower incidence of corpus uteri, oesophagus, and colon cancer. At least 50% or higher mortality among rural women was observed for cervical, liver, gallbladder, and mouth cancer. About 70% lower incidence and 90% lower mortality were reported for ovarian cancer in rural women. Significantly higher mortality for breast cancer was observed in rural women, despite about 20% lower incidence. (Table 2)

## Discussion

The growing burden of cancer and the required continuum of care are facing inequalities and inequities around the world, and one such example is the rural-urban disparity in cancer [17]. Rural residence, though a simple variable, encapsulates a complex surrogate for several potential explanatory factors like access to care, lifestyle, environmental exposure, and various socioeconomic factors [18]. Disparity in cancer outcomes due to rurality is well documented [3, 14, 19, 20]. Moreover, rapid advances in cancer care will further widen the disparity in outcomes for rural patients without directed effort to understand and address barriers to high-quality care in these regions [6]. Understanding the context of this disparity will reveal the specific needs of the population [18], especially for resource-constrained countries like India, which has the largest growing population with a predominantly rural background. Through this paper, we analysed the urban-rural disparity in cancer burden and care for over 6,000 patients in Northern India who were registered under the PBCR of the Varanasi district from the Uttar Pradesh state of India.

### Disparities in the age distribution

We observed that the adult cancer patients aged 45 years and above significantly belonged to urban regions of the district, while the younger patients (aged 15–29 years) were from rural backgrounds. The significantly higher proportion of cancer among younger rural patients can be partly explained by tobacco use, which accounts for approximately 30% of cancers in India [20]. Furthermore, Global Adult and Youth Tobacco Surveys (GATS and GYTS) have reported an early age of tobacco initiation and a higher prevalence of tobacco use, especially in rural populations [21, 22]. Inadequate services for tobacco and alcohol cessation counselling in rural areas exacerbate this problem [23]. Fully-functional adolescent health clinics are the need of the hour and should offer habit cessation counselling as well as screening for common cancers in young adults.

On the other hand, increasing age itself is an independent risk factor for cancer and better access to health care in urban regions is contributing to increased life expectancy, and thereby an increased elderly population in the urban regions [24]. This can somewhat explain the significantly higher proportion of cancer in elderly urban patients. Also, poor access to diagnostic facilities, especially for the rural elderly population, can be another explanation for this distribution. We observed a significantly higher proportion of cancers from O&U among the rural patients, which further highlights this diagnostic disparity. It is therefore imperative to expand the existing facilities in urban areas given the high burden of cancer and simultaneously establish and strengthen facilities for cessation counselling, diagnosis, and treatment in underserved rural areas. Measures such as telemedicine, mobile screening units, mobile health applications, etc. should also be taken to address the barriers to accessing the facilities by the elderly population in both urban and rural areas.

### Disparities in the socio-cultural distribution

We observed a significant difference in the religion of patients; where compared to the rural patients, who were predominantly Hindu, the urban patients belonged to other religions. Our finding is supported by the Indian Census, which reported that religious minorities tend to migrate and live in urban areas for social security [25, 26]. On the other hand, the rural patients predominantly spoke the local vernacular language compared to urban patients, who could communicate in the common Hindi language. Language and cultural barriers to cancer treatment and symptom management have been reported among rural patients with cancer [27]. Patient navigation systems can help overcome this linguistic and cultural barrier [28]. 

There was a significant difference in the type of profession among the patients, where the proportion of professionals and semi-professionals was significantly higher in urban patients while farming, un-skilled, semi-, and skilled professions were predominant in rural patients. Previous studies have reported the success of workplace screening in urban populations and community screening in rural populations and future cancer control policies should implement screening strategies accordingly [29, 30]. 

The educational and socio-economic status were significantly higher in the urban patients, confirming the prevalent socio-economic disparity in the urban-rural population. Educational and socio-economic status are important factors associated with better health literacy, health-seeking behaviour, screening participation and adherence, early stage at diagnosis, compliance with treatment, and follow-up thereby resulting in an overall better survival of the cancer patients. This disparity in accessing cancer care is further worsened in rural populations because of the large proportion of the uninsured population, high out-of-pocket expenditure, and avoidance of seeking care, as many are daily wagers and face illness-related unemployment and increased travel time to access healthcare facilities [31]. 

These socio-demographic factors also influence lifestyle, dietary, behavioural, and environmental factors, as well as healthcare-seeking behaviour and treatment compliance, all of which are decisive entities for cancer survival. It is important to acknowledge urban-rural variability in these factors while designing cancer control programmes. Additionally, realising the spirit of universal health coverage for cancer care is vital to bridge the divide and prevent the resulting impoverishment among already poor and marginalised rural patients with cancer.

### Disparities in cancer burden

The overall incidence of cancer in rural areas was lower compared to urban areas, but mortality was higher in rural areas, especially among women. Our findings align with previous national studies. In rural cancer registries (Barshi), the AAIR is nearly one-third of urban PBCRs [32]. Another study noted that the AAIR in urban Punjab PBCRs (Chandigarh and SAS Nagar) is almost twice that in rural PBCRs (Mansa and Sangrur) [19]. Similar trends are observed in Nepal, where the urban (Kathmandu) registry showed 1.6 times higher AAR among males and 1.9 times higher AAR among females in comparison to the rural (Rukum) registry [33]. Conversely, a study in China revealed significantly higher AAIR in rural men, primarily attributed to oesophageal cancer [14]. Developed countries, like those in North America, consistently reported higher all-site incidence rates in urban populations compared to rural ones [34]. 

A complex interplay of rurality with sociodemographic, lifestyle, dietary, behavioural, and environmental factors that affect the screening participation, incidence, and prognosis of the disease is seldom recognised and addressed. Additionally, rural women face several cultural and social barriers, which further aggravate the misery [3, 35]. This was evident from our findings, where out of the three preventable cancers among women for which screening is recommended in the National programme, two (oral and cervical) had a higher incidence and all three (oral, breast, and cervical) had a higher mortality among rural women. Previous Indian studies have highlighted the inequalities in socioeconomic factors and healthcare utilisation concerning cancer screening in urban and rural populations [5, 36, 37]. Given the above findings, cancer awareness generation and screening activities must acknowledge the dissimilar socio-demographic background characteristics of urban and rural populations and design strategies accordingly.

Furthermore, various healthcare-related factors such as (i) poor access to cancer treatment facilities, (ii) greater likelihood to receive treatment at smaller hospitals, (iii) lower probability of guideline-concordant treatment practices, (iv) lack of genomic testing and staging, (v) disparity in cancer treatment modalities and quality, (vi) treatment attrition, (vii) significant shortage of specialists, (viii) limited supportive and rehabilitative resources, and (ix) inadequate cancer care navigation are more pronounced in rural regions and contribute to higher mortality [39, 39]. Healthcare services in urban areas of India generally receive a larger share of public resources, resulting in lower rural health infrastructure investment coupled with issues of ill management, staff absenteeism, and poor capacity-building efforts [3, 38, 39]. 

### Disparities in the leading cancer sites

We observed a significant difference in the standardised rate ratios for the site-specific cancers for rural and urban patients. Similar observations have been reported from previous cancer registries of India, Nepal, China, and the United States (US) [14, 19, 33, 40]. The significantly higher AAR for liver cancer in rural patients can be partially attributed to a greater prevalence of alcohol use in the rural Indian population. This is compounded by an earlier age at the onset of alcohol consumption, frequent episodes of heavy drinking, and the consumption of non-brewed alcoholic beverages [23]. Also, a greater prevalence of hepatitis B infection and a similar prevalence of non-alcoholic fatty liver disease, which are known risk factors for liver cancer, have been reported among rural Indian residents compared to the urban population [41]. The National Cancer Registry Programme has also reported that liver cancer was highest in the northeastern cancer registries (Papumpasre, West Arunachal, Aizwal, Mizoram) [11] which have a predominantly rural population (81.64%) [42] Similarly, China [14] and US [43] have also reported higher AAR for liver cancer in their rural populations.

Significantly higher AAR of gallbladder cancer in both male and female rural patients can be partly explained by higher mustard oil consumption, the prevalence of cholelithiasis, chronic typhoid infection, and the consumption of snails, which are often contaminated with liver flukes [44, 45]. Arsenic in groundwater has recently been linked to gallbladder cancer, and untreated groundwater consumption is more prevalent in rural areas than urban areas, which might further explain the rural predominance of gallbladder cancer [46]. Several studies from India (cancer registries and case-control studies) have reported rural background as a risk factor for gallbladder cancer [44, 47]. However, studies from countries such as Nepal [33] and Chile [48] have reported urban predominance for gallbladder cancer, which has been explained by the higher prevalence of gallstones, obesity, and hormone use in their urban regions [33, 48]. 

We observed significantly higher incidence rates for colon and oesophageal cancer in both men and women with urban backgrounds compared to their rural counterparts. Higher prevalence of obesity, inadequate physical activity, salt and red meat consumption, diabetes, and low consumption of fibre among urban residents could partly decipher the higher incidence rates in the urban population [49]. Our findings are in line with studies from India that have reported a rising trend in registries established in metropolitan regions (predominantly urban population) such as Delhi, Chennai, Mumbai, and Banglore [50], and studies from China [14, 51]. In contrast, studies from the US show a rural preponderance for colon cancer, which has been attributed to higher red meat consumption, obesity, a lack of physical activity, and lower cancer screening adherence in their rural populations [34, 52]. The urban preponderance of oesophageal cancer observed in our study can be attributed to the increased prevalence of gastroesophageal reflux disease, low fruit and vegetable intake, and obesity, coupled with prevalent tobacco and alcohol use in urban areas [49, 53]. However, Indian registries from another northern state (Punjab) reported a higher AAR of oesophageal cancer in rural registries (Mansa, Sangrur) in comparison to urban registries (SAS Nagar, Chandigarh) [19]. In addition, studies from China also reported a higher preponderance of oesophageal cancer in the rural population [14]. This distinction underscores the heterogeneity in the prevalence of key risk factors, namely tobacco smoking, alcohol consumption, and dietary factors, across intra- and inter-country regions.

We observed that trachea, bronchus, and lung cancer incidence rates were higher in rural patients, which could be explained by indoor air pollution due to biomass burning, exposure to second-hand smoke at home, and tobacco and beedi smoking, which are more prevalent in rural regions of Uttar Pradesh than urban areas [54, 55]. However, our findings are in contrast with rural registries from other states such as Maharashtra (Barshi) and Punjab (Mansa and Sangrur), which reported lower AAR for lung cancer, and urban registries (Chandigarh, SS Nagar, Trivandrum, Chennai, and Delhi), which reported higher incident rates [18, 54]. In addition, several urban registries from eastern African countries (Malawi, Zimbabwe, Uganda, and Kenya) have also reported a high burden of lung cancer [56]. Our finding is in line with registries from Korea [57], China [14], and the US [34], which reported rural predispositions for lung cancer incidence. These diverse observations underscore how various factors, including smoking, indoor and outdoor air pollution, and the utilization of lung cancer screening, interact in different contexts, leading to the urban-rural disparity in lung cancer incidence.

Both penile cancer in men and cervical cancer in women share some of the risk factors, including infection with the Human Papilloma Virus, increasing age, poor hygiene, tobacco use, multiple sexual partners, low education, and socio-economic status. Most of these factors are predominant in rural areas, thereby explaining the high rates of these cancers in rural patients in our study [58, 59]. Cervical cancer predominance in rural women has been unanimously reported in several registry studies from India [19, 60], Nepal [33], Sub-Saharan Africa [61], China [51] and the US [62]. Despite a lower incidence of prostate cancer, rural men had almost similar cancer-related mortality as urban patients, which is worrisome and could be due to a wide urban-rural gap in screening as well as treatment facilities and modalities. Previous Indian research has shown lower screening rates among rural patients, and rural patients with prostate cancer are less likely to receive definitive treatment than their urban counterparts [63–65]. Systematic reviews, which mostly used data from high-income countries, showed that rural-urban differences in prostate cancer incidence and mortality were confirmed. It was found that while incidence was higher in urban men, mortality was higher in rural men. This was partly because of the systemic barriers that made it take longer for men to get diagnosed and treated for prostate cancer [66, 67]. 

A negative rural-urban risk difference in the incidence of endometrial, breast, and ovarian cancer can be attributed to a relatively greater prevalence of risk factors like obesity, metabolic syndrome, nulli-and-low parity, late pregnancy, infertility, use of hormones, early age at menarche, and poor lifestyles like inadequate physical activity, a high-fat diet, and alcohol and tobacco use in urban areas than in rural regions [68]. Our findings are in line with the observations from other Indian PBCRs where breast cancer was the leading cancer in registries of urban agglomerations [19] and cervix cancer was the leading cancer in rural registries like Barshi, Mizoram, Tripura, Pasigat, Nagaland, Cachar, Osmanabad, and Beed [68]. The urban preponderance of these women’s cancers associated with a hormonal etiology has been reported in several studies from Nepal [33], Egypt [69], China [70], and the US [34]. 

We observed that, despite significantly lower breast cancer incidence in rural women, higher mortality was observed in them compared to their urban counterparts. This signifies the rural-urban disparities in the early detection of breast cancer, delayed care seeking, and treatment initiation. Previous Indian studies reported that rural women are less likely to get screened and more likely to present at late stages of breast cancer compared to their urban counterparts [35, 71]. Furthermore, the significantly higher incidence of breast, endometrial, ovarian, and colon cancers in urban female patients hints towards further research for understanding the genetic predisposition and genetic screening and counselling.

We observed that mouth cancer was the predominant cancer in our study population. Surprisingly, despite well-documented higher tobacco use in rural parts of India and Uttar Pradesh [21, 22], we observed a statistically significant higher incidence and mortality of mouth cancer in urban men. Previous studies comparing the incidence rates of oral cancer have also shown significantly higher incidence in urban PBCRs compared to rural PBCRs [72]. Additionally, analysis of a national representative survey also reported higher rates of tobacco-related cancer deaths in urban than rural men [35]. This can be explained to a certain extent by several factors. Firstly, poor cancer screening coverage was reported in the national survey [63] where the oral cancer screening rates were lower in the rural population, thus reflecting the impact of poor screening coverage on the cancer incidence distribution. The second explanation can be the significantly higher proportion of the elderly in our urban study population, and as discussed previously, age itself is an important independent risk factor for carcinogenesis. Thirdly, studies from India have reported that most of the oral cancers detected in rural populations are in advanced stages with poor 5-year survival [38, 73]. Previously, a review on oral cancer burden in India reported variations in the AAR of oral cancer in rural men across many registries. One explanation cited was the lack of transportation, which hinders seeking diagnosis and care in rural populations [73]. Therefore, this contradicting finding from our study underscores the rural-urban disparity in the early detection of oral cancer in this region. Registry from China [14] reported higher AAR in urban men while the North American Registries [34] reported higher incidence in rural men, and both attributed this difference to the higher tobacco consumption in their urban and rural populations, respectively.

### Disparities in the cancer care continuum

We observed a smaller number of cancer confirmations through only death certificates (DCO), where no other clinical records of the patients were available, and these DCO cases were mostly seen in urban patients. Cancer confirmation by verbal autopsy constituted a relatively larger portion of cancer registrations and was seen mostly in rural patients. These findings reflect poor record maintenance, weak medical certification of the cause of death, and various challenges associated with cancer registration in the study region, especially in the rural population compared to the urban population [7]. Furthermore, the proportion of urban patients who had microscopic verification for cancer confirmation (71.1%) was higher than that of rural patients (57.7%). In addition, we observed a higher proportion of incomplete treatment among rural patients with cancer. This can be partly attributed to the reliance on alternative systems of medicine, cancer fatalism, and poor health and cancer literacy, which are the pragmatic challenges present predominantly in the rural parts of our study settings. We observed that definitive treatment, including multi-modality treatment, was significantly higher in urban patients. A recent Indian survey reported that the average number of patients with cancer attending public outpatient service management was higher in urban areas. Moreover, the survey reported a lower proportion of facilities for cancer screening and inpatient and outpatient cancer management services in rural areas compared to their urban counterparts [23]. Hence, it is imperative to engage multi-sectoral stakeholders to develop patient advocacy networks, especially for rural resource-deprived regions, to improve healthcare seeking and compliance as well as prevent treatment attrition.

A significantly higher proportion of rural patients were receiving palliative care than their urban counterparts. The lack of organized screening, diagnostic, and referral facilities in rural areas, resulting in delayed diagnosis, can explain this difference. Strategies to improve their accessibility and affordability will aid in the early detection and downstaging of cancers. Furthermore, 138 Indian organizations providing hospice and palliative care services are concentrated in large cities and regional cancer centres, except for Kerala. Therefore, it necessitates long-distance journeys for rural patients to access palliative care in urban settings [74]. Thus, there is a pressing need to introduce palliative care at the primary health care level.

### Limitations

This was a newly established PBCR; hence, we had limited follow-up information to analyse and describe 5-year survivals for leading cancer sites. In addition, the case ascertainment completeness indices such as the proportion of microscopic verification, DCO, and AAIR of childhood cancers reflected under-registration by 10–20% within the different blocks of the district, especially among the rural, elderly, and paediatric (especially girl child) populations, partly due to the disparity in accessibility of the services [75]. Since we could utilise only the incidence-based data, information related to some important variables such as staging of cancer, health insurance status, co-morbidities, and risk factors such as lifestyle, dietary behaviour, and environmental factors could not be evaluated to explain the observed disparities. Lastly, we cannot generalise these observations to reflect the extent of the nationwide rural-urban disparity in cancer incidence and patterns.

### Strengths

To the best of our knowledge, this is the first of its kind study from India that has analysed the risk of site-specific cancers and the disparities in the social-demographic characteristics of patients with cancer, cancer burden, and patterns among the rural and urban populations. Owing to the longitudinal data, we could analyse the urban-rural disparity in terms of overall and site-specific cancer mortality.

## Conclusion

Low- and middle-income countries face two distinct challenges when it comes to cancer prevention and control: in their urban areas, unhealthy lifestyle changes that are linked to an increased risk of cancer are being observed, and in their rural areas, a lack of access to healthcare leads to delayed diagnosis and poor survival. Based on these findings, we recommend context-specific interventional programmes targeting risk-factor modifications, cancer awareness, early detection, and accessibility to diagnosis and care. These observed geographical and social variations for the specific cancer sites warrant further research to understand the causation of cancer. Our study reflects this distinctness in cancer burden and pattern, especially for the female population, and calls for a re-evaluation of cancer control strategies that are tailor-made with an understanding of urban-rural disparities. We are further planning to study the completeness of the cancer registry in the coming years.

### Electronic supplementary material

Below is the link to the electronic supplementary material.

**Supplementary Material 1:** Additional file of data quality indices of Varanasi cancer registry, socio-demographic profile of patients with cancer and the incident rate and cumulative risk for leading cancer sites

## Data Availability

The datasets generated and/or analyzed to support this study’s findings are contained within the Varanasi population-based cancer registry, but are not publicly available due to confidentiality, security, and ownership matters. They may be available from the corresponding author upon reasonable request.

## References

[1] Shah SC, Kayamba V, Peek RM (2019). Cancer Control in Low-and Middle-Income countries: is it time to consider screening?. J Glob Oncol.

[2] Dhillon PK, Mathur P, Nandakumar A (2018). The burden of cancers and their variations across the States of India: the global burden of Disease Study 1990–2016. Lancet Oncol.

[3] Bhatia S, Landier W, Paskett ED (2022). Rural-urban disparities in Cancer outcomes: opportunities for Future Research. J Natl Cancer Inst.

[4] Ministry of Rural Development. Shyama Prasad Mukherji Rurban Mission. National Portal of India. [cited 2023 Oct 4]. Available from: https://www.india.gov.in/spotlight/shyama-prasad-mukherji-rurban-mission.

[5] Banerjee S (2021). Determinants of rural-urban differential in healthcare utilization among the elderly population in India. BMC Public Health.

[6] Shastri SS (2018). Cancer trends and disparities in India: data needs for providing equitable cancer care. Lancet Oncol.

[7] Khanna D, Budukh A, Sharma P (2023). Role of verbal autopsy in cancer registration: a mixed-methods study from the population-based cancer registry of Northern India. Trop Med Int Health.

[8] Behera P, Patro BK (2015). Population Based Cancer Registry of India– the challenges and opportunities. Asian Pac J Cancer Prev.

[9] Ministry of Health and Family Welfare. Government of India. National Health Policy 2017. [cited 2023 Oct 4]. Available from: https://main.mohfw.gov.in/sites/default/files/9147562941489753121.pdf.

[10] Budukh AM, Pradhan S, Singh VB (2023). Cancer pattern in Varanasi district from Uttar Pradesh state of India, a foundation for cancer control based on the first report of the population-based cancer registry. Indian J Cancer.

[11] Report of National Cancer Registry Programme– Indian Council of Medical Research.:2012–2016, Bengaluru. 2020. [cited 2023 Nov 12]. Available from https://ncdirindia.org/All_Reports/PBCR_Annexures/Default.apx.

[12] International Incidence of Childhood Cancer Volume III: International Agency for Research on Cancer (IARC)., World Health Organization (WHO). [cited 2023 Oct 4] Available from https://iicc.iarc.fr/results/introduction/qualityindicators.pdf.

[13] Bhagat RB, Mohanty S (2009). Emerging pattern of urbanization and the contribution of migration in urban growth in India. Asian Popul Stud.

[14] Yuan S, Xie SH (2021). Urban-rural disparity in cancer incidence in China, 2008–2012: a cross-sectional analysis of data from 36 cancer registers. BMJ Open.

[15] Boyle P, Parkin DM, International Agency for Cancer Research. Statistical methods for registries. 1991. [cited 2023 Oct 4]. Available from: https://scholar.google.com/scholar_lookup?journal=Cancer+Regist+Princ+Methods&title=Statistical+methods+for+registries&author=P+Boyle&author=D+Parkin&volume=95&publication_year=1991&pages=126-158&.

[16] Bursac Z, Gauss CH, Williams DK, Hosmer DW (2008). Purposeful selection of variables in logistic regression. Source Code Biol Med.

[17] World Health Organization. World Cancer Day: closing the care gap. 2023. [cited 2023 Oct 4]. Available from: https://www.who.int/news/item/03-02-2022-world-cancer-day-closing-the-care-gap.

[18] Thompson JA, Chollet-Hinton L, Keighley J (2021). The need to study rural cancer outcome disparities at the local level: a retrospective cohort study in Kansas and Missouri. BMC Public Health.

[19] Thakur JS, Budukh A, Kapoor R (2017). Urban–rural differences in cancer incidence and pattern in Punjab and Chandigarh: findings from four new population-based cancer registries in North India. Int J Noncommunicable Dis.

[20] Factsheet. - National Cancer Registry Programme– 2020 (ICMR-NCDIR), Bengaluru, India. 2020. [cited 2023 Oct 4] Available from https://ncdirindia.org/All_Reports/Report_2020/default.aspx.

[21] Tata Institute of Social Sciences (TISS)., Mumbai and Ministry of Health and Family Welfare, Government of India. Global Adult Tobacco Survey GATS 2 India, 2016-17 [cited 2023 Oct 4] Available from https://ntcp.mohfw.gov.in/assets/document/surveys-reports-publications/Global-Adult-Tobacco-Survey-Second-Round-India-2016-2017.pdf.

[22] International Institute for Population Sciences and Ministry of Health and Family Welfare, Government of India. Global Youth Tobacco Survey GYTS 4 India, 2019. [cited 2023 Oct 4]. Available from https://ntcp.mohfw.gov.in/assets/document/National_Fact_Sheet_of_fourth_round_of_Global_Youth_Tobacco_Survey_GYTS-4.pdf.

[23] ICMR-NCDIR, National Noncommunicable Disease Monitoring Survey (NNMS) 2017–18, Bengaluru, India. https://www.ncdirindia.org/nnms/.

[24] Ministry of Health and Family Welfare. Average Life Expectancy. Press Information Bureau, Delhi. 2020. [cited 2023 Oct 4] Available from https://pib.gov.in/PressReleasePage.aspx?PRID=1606209.

[25] RGI releases Census 2011 data on Population by Religious Communities., 2015. [cited 2023 Jan 20] https://pib.gov.in/newsite/printrelease.aspx?relid=126326.

[26] Gaikwad N, Nellis G. The majority-minority divide in attitudes toward internal migration: evidence from Mumbai. Am J Polit Sci. 2017;456–72. 10.1111/ajps.12276.

[27] Itty TL, Hodge FS, Martinez F (2014). Shared and unshared barriers to cancer symptom management among urban and rural American indians. J Rural Health.

[28] Chan RJ, Milch VE, Crawford WF (2023). Patient navigation across the cancer care continuum: an overview of systematic reviews and emerging literature. CA Cancer J Clin Online.

[29] Birur P, Patrick S, Bajaj S (2018). A novel mobile-health approach to early diagnosis of oral cancer. J Contemp Dent Pract.

[30] Basu P, Mahajan M, Patira N (2019). A pilot study to evaluate home-based screening for the common non-communicable diseases by a dedicated cadre of community health workers in a rural setting in India. BMC Public Health.

[31] Shruti T, Khanna D, Khan A (2023). Status and determinants of early detection of oral premalignant and malignant lesions in India. Cancer Control.

[32] Mathur P, Sathishkumar K, Chaturvedi M, Icmr-Ncdir-Ncrp Investigator Group (2020). Cancer statistics, 2020: report from national cancer registry programme, India. JCO Glob Oncol.

[33] Subedi R, Budukh A, Chapagain S (2021). Differences in cancer incidence and pattern between urban and rural Nepal: one-year experience from two population-based cancer registries. Ecancermedicalscience.

[34] Zahnd WE, James AS, Jenkins WD (2018). Rural–urban differences in cancer incidence and trends in the United States. Cancer Epidemiol Biomarkers Prev.

[35] Dikshit R, Gupta PC, Ramasundarahettige C (2012). Cancer mortality in India: a nationally representative survey. Lancet.

[36] Negi J, Nambiar D (2021). Intersectional social-economic inequalities in breast cancer screening in India: analysis of the National Family Health Survey. BMC Womens Health.

[37] Mishra R, Monica M (2020). An epidemiological study of cervical and breast screening in India: district-level analysis. BMC Women’s Health.

[38] Banavali SD (2015). Delivery of cancer care in rural India: experiences of establishing a rural comprehensive cancer care facility. Indian J Med Paediatr Oncol.

[39] Pramesh CS, Badwe RA, Borthakur BB (2014). Delivery of affordable and equitable cancer care in India. Lancet Oncol.

[40] National Cancer Institute. GIS Portal for Cancer Research. Rural-Urban Disparities in Cancer. [cited 2023 Oct 4] Available from https://gis.cancer.gov/mapstory/rural-urban/index.html.

[41] Kumar D, Peter RM, Joseph A (2023). Prevalence of viral hepatitis infection in India: a systematic review and meta-analysis. J Educ Health Promot.

[42] Census of India– 2011. Census India Library. Office of the Registrar General and Census Commissioner, Government of India. [2019 cited Nov 26]. Available from: http://censusindia.gov.in/DigitalLibrary/MFTableSeries.aspx.

[43] Mezzacappa C, Rossi R, Jaffe A, et al. Community-level factors associated with hepatocellular carcinoma incidence and mortality: an observational registry study. Cancer Epidemiol Biomarkers Prev. 2024;Jan 3(OF1–9). 10.1158/1055-9965.epi-23-0902. 10.1158/1055-9965.EPI-23-0902 PMC10872555 38059831

[44] Dutta U, Bush N, Kalsi D (2019). Epidemiology of gallbladder cancer in India. Chin Clin Oncol.

[45] Mhatre S, Rajaraman P, Chatterjee N (2020). Mustard oil consumption, cooking method, diet and gallbladder cancer risk in high-and low‐risk regions of India. Int J Cancer.

[46] Shridhar K, Krishnatreya M, Sarkar S (2023). Chronic exposure to drinking Water Arsenic and Gallbladder Cancer Risk: preliminary evidence from endemic regions of India. Cancer Epidemiol Biomarkers Prev.

[47] Barbhuiya MA, Singh TD, Poojary SS (2015). Gallbladder cancer incidence in Gwalior district of India: five-year trend based on the registry of a regional cancer center. Indian J Cancer.

[48] Bertran E, Heise K, Andia ME (2010). Gallbladder cancer: incidence and survival in a high-risk area of Chile. Int J Cancer.

[49] Lewandowska A, Rudzki G, Lewandowski T (2022). Risk factors for the diagnosis of colorectal cancer. Cancer Control.

[50] Shakuntala TS, Krishnan SK, Das P (2022). Descriptive epidemiology of gastrointestinal cancers: results from National Cancer Registry Programme, India. Asian Pac J Cancer Prev.

[51] Li X, Deng Y, Tang W (2018). Urban-rural disparity in cancer incidence, mortality, and survivals in Shanghai, China, during 2002 and 2015. Front Oncol.

[52] Zahnd WE, Gomez SL, Steck SE (2021). Rural-urban and racial/ethnic trends and disparities in early‐onset and average‐onset colorectal cancer. Cancer.

[53] Zhang M, Hou ZK, Huang ZB (2021). Dietary and lifestyle factors related to gastroesophageal reflux disease: a systematic review. Ther Clin Risk Manag.

[54] Nath A, Sathishkumar K, Das P (2022). A clinicoepidemiological profile of lung cancers in India–results from the National Cancer Registry Programme. Indian J Med Res.

[55] Tata Institute of Social Sciences (TISS)., Mumbai and Ministry of Health and Family Welfare, Government of India. Global Adult Tobacco Survey GATS 2 India 2016-17. Fact Sheet (State Level) [cited 2024 Jan 20] Available from GATS2 Uttar Pradesh https://ntcp.mohfw.gov.in/surveys_reports_publications.

[56] Cheng ML, Zhang L, Borok M (2015). The incidence of oesophageal cancer in Eastern Africa: identification of a new geographic hot spot?. Cancer Epidemiol.

[57] Song HN, Go SI, Lee WS (2016). Population-based regional cancer incidence in Korea: comparison between urban and rural areas. Cancer Res Treat.

[58] Chaux A, Netto GJ, Rodríguez IM (2013). Epidemiologic profile, sexual history, pathologic features, and human papillomavirus status of 103 patients with penile carcinoma. World J Urol.

[59] Kashyap N, Krishnan N, Kaur S (2019). Risk factors of cervical cancer: a case-control study. Asia Pac J Oncol Nurs.

[60] Sathishkumar K, Vinodh N, Badwe RA (2021). Trends in breast and cervical cancer in India under National Cancer Registry Programme: an age-period-cohort analysis. Cancer Epidemiol.

[61] Jedy-Agba E, Joko WY, Liu B (2020). Trends in cervical cancer incidence in sub-saharan Africa. Br J Cancer.

[62] Yu L, Sabatino SA, White MC (2019). Peer reviewed: rural–urban and racial/ethnic disparities in invasive cervical cancer incidence in the United States, 2010–2014. Prev Chronic Dis.

[63] Government of India, Ministry of Health & Family Welfare. National Family Health Survey (NFHS-5) 2019-21. (2021) Government of India, Ministry of Health & Family Welfare, International Institute for Population Sciences. [cited October 6 2023]. http://rchiips.org/nfhs/factsheet_NFHS-5.shtml.

[64] Pandit AA, Patil NN, Mostafa M (2023). Rural–urban disparities in patient care experiences among prostate Cancer survivors: a SEER-CAHPS study. Cancers.

[65] Budukh AM, Thakur JS, Dora TK (2023). Overall survival of prostate cancer from Sangrur and Mansa cancer registries of Punjab state, India. Indian J Urol.

[66] Afshar N, English DR, Milne RL (2019). Rural–urban residence and cancer survival in high-income countries: a systematic review. Cancer.

[67] Obertova Z, Brown C, Holmes M (2012). Prostate cancer incidence and mortality in rural men–a systematic review of the literature. Rural Remote Health.

[68] Chaturvedi M, Sathishkumar K, Lakshminarayana SK (2022). Women cancers in India: incidence, trends and their clinical extent from the National Cancer Registry Programme. Cancer Epidemiol.

[69] Dey S, Hablas A, Seifeldin IA et al. Urban–rural differences of gynaecological malignancies in Egypt (1999–2002). BJOG.2010;117:348– 55. 10.1111/j.1471-0528.2009.02447.x. 10.1111/j.1471-0528.2009.02447.x PMC4291060 20015310

[70] Jiang X, Tang H, Chen T (2018). Epidemiology of gynecologic cancers in China. J Gynecol Oncol.

[71] Nagrani RT, Budukh A, Koyande S (2014). Rural urban differences in breast cancer in India. Indian J Cancer.

[72] Sharma S, Satyanarayana L, Asthana S (2018). Oral cancer statistics in India on the basis of first report of 29 population-based cancer registries. J Oral Maxillofac Pathol.

[73] Coelho KR (2012). Challenges of the oral cancer burden in India. J Cancer Epidemiol.

[74] Khosla D, Patel FD, Sharma SC (2012). Palliative care in India: current progress and future needs. Indian J Palliat Care.

[75] Raza SA, Jawed I, Zoorob RJ (2020). Completeness of Cancer Case Ascertainment in International Cancer registries: exploring the issue of gender disparities. Front Oncol.

[76] National Ethical Guidelines for Biomedical and Health Research Involving Human Participants. Indian Council of Medical Research 2017. [cited 2023 Dec 27]. Available from https://ethics.ncdirindia.org/asset/pdf/ICMR_National_Ethical_Guidelines.pdf.

[77] Revised Common Rule Consideration for Use of State Mandated Central Cancer Registry Data: Guidance, Examples, and Q&A. National American Association of Central Cancer Registries. 2021 [cited 2023 Dec 27]. Available from https://www.naaccr.org/wp-content/uploads/2021/03/Revised-Common-Rule-QA-for-Registries_Final_3.26.21.pdf.

