# Supplementary material for: Rural-urban disparity in cancer burden and care: findings from an Indian cancer registry
Source: BMC Cancer. 2024 Mar 6;24:308. doi: 10.1186/s12885-024-12041-y (PMC10916062; doi:10.1186/s12885-024-12041-y)
Supplement: Supplementary file 1 — Supplementary Material 1: Additional file of data quality indices of Varanasi cancer registry, socio-demographic profile of patients with cancer and the incident rate and cumulative risk for leading cancer sites [file 12885_2024_12041_MOESM1_ESM.docx]

**Additional file of data quality indices of Varanasi cancer registry, socio-demographic profile of patients with cancer and the incident rate and cumulative risk for leading cancer sites.**

**Supplementary Table 1. Data quality indices for completeness of cancer case ascertainment, Varanasi PBCR, 2017-2019 (N=6721)**

| **Data quality indices** | **Male** | **Female** | **Overall** |
| --- | --- | --- | --- |
| Mortality-incidence (MI) ratio* | 65.2% | 61.1% | 63.0% |
| Microscopic verification | 65.3% | 64.2% | 64.8% |
| Death Certificate Only (DCO)/others (verbal autopsy)$ | 17.2% | 17.0% | 17.1% |
| Unspecified subsite  (ICD- (C26, C39, C48, C75, C76, C77, C78, C79, C80, C97) | 4.2% | 2.7% | 3.5% |
| AAIR of childhood cancer per million (0-14 years) # | 63.7 | 27.9 | 47.0 |

*Comparable with other Indian rural PBCRs^11^

$ In the first year of the cancer registry, DCO cases were more than 20% due to an inadequate death registration system which has gone down to 14% in the year 2018-2019 due to the efforts by the cancer registry and the establishment of more tertiary cancer centres in the district.

# AAIR less than 60 per million suggests childhood cancer under-registration^12^

**Supplementary Table 2. Socio-demographic profile of adult patients with cancer (N=6565).**

| **Variables** | **Frequency** | **Percentage** |
| --- | --- | --- |
| **Age groups (in completed years)** |  |  |
| 15-29 | 387 | 5.9 |
| 30-44 | 1330 | 20.3 |
| 45-59 | 2432 | 37.0 |
| ≥60 | 2416 | 36.8 |
| **Sex** |  |  |
| Male | 3670 | 55.9 |
| Female | 2895 | 44.1 |
| **Educational qualification** |  |  |
| Illiterate | 1370 | 20.9 |
| Literate | 1471 | 22.4 |
| Upto secondary | 2626 | 40.0 |
| Senior secondary or higher | 1021 | 15.6 |
| No information/unknown | 77 | 1.2 |
| **Residence** |  |  |
| Rural | 3220 | 49.0 |
| Urban | 3345 | 51.0 |
| **Religion** |  |  |
| Hindu | 5774 | 88.0 |
| Others | 791 | 12.0 |
| **Mother tongue** |  |  |
| Hindi | 6099 | 92.9 |
| Others | 466 | 7.1 |
| **Occupation** |  |  |
| Professional, semi-professional, clerical, government employee, private employee | 1143 | 17.4 |
| Farmer, skilled worker, semi-skilled worker, unskilled worker, others | 2699 | 41.1 |
| Unemployed, student, house-wife | 2678 | 40.8 |
| No information | 45 | 0.7 |
| **Socio-economic status** |  |  |
| Upper and upper middle | 916 | 14.0 |
| Lower middle | 2253 | 34.3 |
| Lower | 2901 | 44.2 |
| No information/unknown | 495 | 7.5 |
| **Primary site large organs system** |  |  |
| Lip, oral cavity, and pharynx (C00-C14) | 1747 | 26.6 |
| Digestive organs (C15-26) | 1494 | 22.8 |
| Respiratory system and intrathoracic organ (C30-39) | 411 | 6.3 |
| Bone, joints, cartilage, skin, connective and other soft tissues (C40-41, C44, C49) | 224 | 3.4 |
| Hematopoietic, reticuloendothelial, and related lymph nodes (C42, C77) | 476 | 5.1 |
| Peripheral and central nervous system (C47, C69-72) | 120 | 1.8 |
| Thyroid, endocrine glands, and related peritoneum and retroperitoneum (C48, 73-75, 76) | 87 | 3.4 |
| Breast (C50) | 705 | 10.7 |
| Female genital organs (C51-58) | 695 | 10.6 |
| Male genital organs (C60-63) | 207 | 3.2 |
| Urinary tract and other urinary organs  (C64-68) | 171 | 2.6 |
| Other and unspecified (O&U) (C80) | 228 | 3.5 |
| **Basis of diagnosis** |  |  |
| DCO | 36 | 0.5 |
| Clinical | 539 | 8.2 |
| Radiology | 654 | 10.0 |
| Cytology | 694 | 10.6 |
| Histology of primary | 3541 | 53.9 |
| Verbal autopsy | 1101 | 16.8 |
| **Treatment** |  |  |
| Surgery | 530 | 8.1 |
| RT | 186 | 2.8 |
| CT | 1019 | 15.5 |
| Multi-modality | 2498 | 38.1 |
| Other alternative system | 240 | 3.7 |
| Palliative | 1258 | 19.2 |
| No treatment | 330 | 5.0 |
| No information/unknown | 504 | 7.7 |
| **Treatment status** |  |  |
| Complete | 1465 | 22.3 |
| Ongoing | 1381 | 21.0 |
| Not completed | 2712 | 41.3 |
| Not applicable | 330 | 5.0 |
| No information/unknown | 677 | 10.3 |
| **Status** |  |  |
| Alive | 2369 | 36.1 |
| Dead | 4196 | 63.9 |

DCO- death certificate only, RT- Radiotherapy, CT- Chemotherapy

**Supplementary Table 3. Incidence rate (AAR per 100 000) and Cumulative risk (% and 1 in person) of leading cancer sites, Varanasi, India, 2017-2019. (N=6721)**

| **Cancer Sites** | **Urban** | | | **Rural** | | |
| --- | --- | --- | --- | --- | --- | --- |
|  | **Incidence** | **Cum Risk (0-74) %** | **1 in Persons** | **Incidence** | **Cum Risk (0-74) %** | **1 in Persons** |
| **Male** | | | | | | |
| All Sites | 81.7 | 0.0936 | 11 | 60.8 | 0.07152 | 14 |
| Mouth (C03-06) | 24.3 | 0.0260 | 38 | 15.0 | 0.01678 | 60 |
| Tongue (C01-02) | 7.1 | 0.0079 | 126 | 3.9 | 0.00464 | 215 |
| Prostate (C61) | 4.1 | 0.0056 | 180 | 2.8 | 0.00370 | 271 |
| Trachea, bronchus, and lung (C33-34) | 3.5 | 0.0049 | 206 | 3.7 | 0.00464 | 216 |
| Gallbladder (C23-24) | 3.2 | 0.0043 | 233 | 3.5 | 0.00430 | 232 |
| Liver (C22) | 2.7 | 0.0030 | 328 | 4.1 | 0.00509 | 196 |
| **Female** | | | | | | |
| All Sites | 63.2 | 0.07226 | 14 | 59.0 | 0.063064 | 16 |
| Breast (C50) | 15.9 | 0.01779 | 56 | 12.7 | 0.012984 | 77 |
| Cervix uteri (C53) | 6.9 | 0.00872 | 115 | 10.3 | 0.011433 | 87 |
| Gallbladder (C23-24) | 6.2 | 0.00785 | 127 | 8.7 | 0.009569 | 105 |
| Ovary (C56) | 4.5 | 0.00510 | 196 | 3.2 | 0.003001 | 333 |
| Mouth (C03-06) | 2.3 | 0.00285 | 351 | 2.9 | 0.003008 | 332 |
| Liver (C22) | 1.9 | 0.00227 | 440 | 3.0 | 0.003348 | 299 |
